# Anti-PD1/PD-L1 Immunotherapy for Non-Small Cell Lung Cancer with Actionable Oncogenic Driver Mutations

**DOI:** 10.3390/ijms22126288

**Published:** 2021-06-11

**Authors:** Edouard Dantoing, Nicolas Piton, Mathieu Salaün, Luc Thiberville, Florian Guisier

**Affiliations:** 1 Department of Pneumology, CHU Rouen, 76000 Rouen, France; edouard.dantoing@chu-rouen.fr (E.D.); mathieu.salaun@univ-rouen.fr (M.S.); Luc.thiberville@univ-rouen.fr (L.T.); 2 Department of Pathology, CHU Rouen, 76000 Rouen, France; Nicolas.piton@chu-rouen.fr; 3 QuantIF Team, LITIS Lab EA4108, UNIROUEN, Normandie University, 76000 Rouen, France; 4 Inserm CIC CRB 1404, CHU Rouen, 76000 Rouen, France

**Keywords:** non-small cell lung cancer, anti-PD1/PD-L1 immunotherapy, EGFR, ALK, BRAF, MET, HER2, RET, ROS1, oncogenic driver

## Abstract

Anti-PD1/PD-L1 immunotherapy has emerged as a standard of care for stage III-IV non-small cell lung cancer (NSCLC) over the past decade. Patient selection is usually based on PD-L1 expression by tumor cells and/or tumor mutational burden. However, mutations in oncogenic drivers such as *EGFR*, *ALK*, *BRAF*, or *MET* modify the immune tumor microenvironment and may promote anti-PD1/PD-L1 resistance. In this review, we discuss the molecular mechanisms associated with these mutations, which shape the immune tumor microenvironment and may impede anti-PD1/PD-L1 efficacy. We provide an overview of the current clinical data on anti-PD1/PD-L1 efficacy in NSCLC with oncogenic driver mutation.

## 1. Introduction

Lung cancer is the leading cause of cancer-related death worldwide, with an estimated 1.76 million deaths in 2018 (18.4% of total cancer deaths) [1]. Overall lung cancer has a poor prognosis, with 18.6% of patients surviving 5 years [2]. Approximately 80% of lung cancer cases are attributed to cigarette smoking [3], while 10–25% occur in never smokers [4]. Exposure to environmental carcinogens such as asbestos, radon gas, or other forms of pollution are the other main causes [5].

Lung cancer is classified in two major types: small cell lung cancer (SCLC), which accounts for 15–20% of lung cancer patients, and non-small cell lung cancer (NSCLC), comprising the remaining 80–85% [6] and subclassified in three major histological subtypes: adenocarcinoma (40% of all lung cancer cases), squamous cell carcinoma (20% of all lung cancer cases), and large cell carcinoma (LCC) [7]. Adenocarcinoma is the predominant subtype in never smokers [7]. Over the past two decades, genomic studies of large cohorts have unraveled a complex molecular landscape of lung tumors.

Current guidelines for the diagnosis and management of adenocarcinoma include histological subtyping and molecular analysis. In fact, targeted therapies for several oncogenic alterations have been developed and improve patients’ outcomes (Table 1). In stage IV adenocarcinoma patients, *EGFR*, *ALK*, *ROS1*, *BRAF*, *MET*, *RET*, *HER2*, *KRAS*, and *NTRK* are assessed to offer targeted therapy for eligible patients [8,9]. Alterations in these so-called “actionable” oncogenes are usually mutually exclusive, which indicates that these individual genes are capable of driving lung cancer progression.

Since 2015, anti-programmed death 1 (PD1) or anti-programmed death-ligand 1 (PD-L1) immunotherapy has emerged as a gold-standard treatment for first- or second-line treatment of stage IV NSCLC, either in monotherapy or in combination with chemotherapy, after several clinical trials demonstrated their benefits over chemotherapy in second and then first-line treatment (Table 2). In most of these studies, patients whose tumor harbored oncogenic alterations (particularly *EGFR* mutations and *ALK* and *ROS1* rearrangement) were excluded. In fact, efficacy of anti-PD1/PD-L1 immunotherapy was thought to be scarce in *EGFR*-mutated NSCLC. As a result, few clinical data are available in this subset of patients.

Anti-PD1/PD-L1 immunotherapy acts by blocking an inhibitory lymphocyte receptor, PD1, though releasing the anti-tumor immune cytotoxicity [53].

**Table 2:** Results of the main trials evaluating anti-PD1/PD-L1 monotherapy in stage IV NSCLC.

	Histology	PDL1	*n*	ORR (%) *	OS (mo) *	Ref.
**First-line**						
Nivolumab	NSCLC	>5%	271	26 vs. 33	13.7 vs. 13.8	[54]
Pembrolizumab	NSCLC	>50%	154	45 vs. 28	30 vs. 14.2	[55]
	NSCLC	>1%	638	27 vs. 27	16.7 vs. 12.1	[56]
Atezolizumab	NSCLC	>1%	277	38.3 vs. 28.6	20.2 vs. 13.1	[57]
Durvalumab	NSCLC	>25%	369	35.6 vs. 37.7	16.3 vs. 12.9	[58]
Cemiplimab	NSCLC	>50%	283	37 vs. 21	22.1 [17.5-NR] vs. 14.2	[59]
**Second- or third-line**						
Nivolumab	Squamous	All	135	20 vs. 9	9.2 vs. 6	[60]
	Adenocarcinoma	All	292	19 vs. 12	12.2 vs. 9.4	[61]
Pembrolizumab	NSCLC	>1%	344	18 vs. 9.3	10.4 vs. 8.5	[62]
Atezolizumab	NSCLC	All	425	14 vs. 13	13.8 vs. 9.6	[63]

*n*: number of patients in the experimental arm. ORR: objective response rate. OS: overall survival. * comparison of ORR and OS data is given in the following format: experimental arm (anti-PD1/PD-L1) versus standard of care arm (chemotherapy).

Expression of PD-L1 by tumor and immune cells, high tumor mutational burden (TMB), and tumor infiltration by immune cells are key features associated with a better efficacy of anti-PD1/PD-L1 immunotherapy in stage IV NSCLC [64]. By modeling these 3 characteristics, oncogenic driver mutations may impede anti-PD1/PD-L1 efficacy [65]. In this review, we discuss the immune-related parameters associated with actionable oncogenic driver mutations and provide an overview of the current clinical data on anti-PD1/PD-L1 efficacy in NSCLC with such mutations.

## 2. NSCLC Actionable Oncogenic Drivers and the Immune Micro-Environment

### 2.1. PDL1 Expression in NSCLC with Actionable Oncogenic Driver Mutation

Contradictory results have been reported regarding PD-L1 expression in *EGFR*-mutated NSCLC (Table 3). Early reports showed upregulation of PD-L1 in *EGFR*-mutated NSCLC cell lines and animal models [66,67] as well as some patient data [68,69,70]. Noteworthy, PD-L1 assessment in these studies used various non-standardized assays. The most recent studies used clinically validated assays and tested samples from treatment-naïve patients. A pooled analysis of 15 public studies gathering 1050 *EGFR*-mutated NSCLC patients showed that patients with *EGFR* mutations had decreased PD-L1 expression (odds ratio: 1.79, 95% CI: 1.10–2.93; *p* = 0.02) [71]. This was consistent with data from 237 lung adenocarcinomas from The Cancer Genome Atlas [72] and with a recent report on 336 treatment-naïve patients with *EGFR*-mutated NSCLC [73].

In 319 patients with *EGFR*-mutant NSCLC, Cho et al. showed that PD-L1 expression is more prevalent in stage II-IV than in stage I tumors, and in exon 19 deletion than in L858R mutation [74].

When a tumor progresses after EGFR targeted therapy, *EGFR* T790M mutation is found in 50% of cases. Tumors that are T790M negative are more likely to express PD-L1 and patients may have greater benefit from anti-PD1/PD-L1 therapy in this setting [75,76]. In a recent paper, PDL1 expression was found to be higher in EGFR T790M positive after progression on Osimertinib: 5/10 had PD-L1 expression > 1% after progression vs. 0/10 at baseline [77]. Among other EGFR mutations, exon 20 insertions were associated with a higher frequency of PD-L1 expression [78,79].

In 111 NSCLC patients with *MET* exon 14 skipping mutations, Sabari et al. found a higher PD-L1 expression than expected from the above-mentioned studies, with 22%, and 41% having PD-L1 expression of 1–49%, and ≥ 50%, respectively [80]. This result was confirmed in a recent analysis [81]. Nevertheless, the median TMB of *MET* exon 14-altered lung cancers was lower than that of unselected NSCLCs. Similar results were recently reported in two series of 14 and 20 NSCLC patients with *MET* exon 14 skipping mutations [82,83].

Among 122 patients with *HER2*-mutated NSCLC, PD-L1 expression was found to be low, with 13% of patients having PD-L1 expression over 50% [84]. In another study, 1/9 patient had PDL-1 over 50% [85]. Similarly, no tumors had PD-L1 expression over 50% in two other series of 15 and 13 *HER2*-mutated NSCLC patients [82,83]. In the latter series, TMB was ≤5 Mut/Mb in all 13 cases. Recently, two more studies reported data on 13 and 21 HER2-mutated NSCLC patients, respectively, showing that 3/13 had a PD-L1 expression > 50% [79] and 4/21 tumors a PD-L1 expression > 1% [78].

A retrospective cohort of 39 patients with *BRAF*-mutant NSCLC (21 *V600E*- and 18 non-*V600E*) was recently reported, showing that 45% of patients had high PDL-1 expression (>50%) [86]. In this study, TMB was ≥20 Mut/Mb in 25% of *BRAF* V600E tumors but 0% of non-*V600E* mutant tumors. Similar findings were reported in 18 BRAF-mutant NSCLC (9 V600E and 9 non-V600E) [82].

Data for other oncogenic drivers are scarce. In *ALK*-rearranged NSCLC, PD-L1 expression over 50% was reported in 5/19, 4/10, 0/11, and 2/9 tumors [83,87,88,89]. *NTRK* gene fusions in NSCLC may be associated with higher TMB and PD-L1 expression than other molecularly defined subgroups [90]. In *KRAS* G12C mutation NSCLC, PD-L1 expression was reported to be ≥ 1% in 16/40 tumors [91]. Controversial data have been reported for *ROS1* and *RET* rearranged NSCLC [82,83,85,88,92,93,94].

### 2.2. Immunogenicity and Lymphocyte Infiltration of NSCLC with Actionable Oncogenic Driver Mutation

The infiltration of CD8+ T lymphocytes has been found to reduce in *EGFR*-mutated NSCLCs compared to those with *EGFR* WT [71,73,95].

In a study of 336 treatment-naïve *EGFR*-mutated NSCLC, authors also provided evidence for a low immunogenicity of *EGFR*-mutated NSCLC by analyzing the TCGA data and an independent validation cohort of patients [73]. They found that patients with *EGFR* mutation had lower TMB than those with *EGFR* wild-type. More importantly, there was a significant difference in TMB between *EGFR*-sensitive (exon 19Del, L858R, L861Q, G719X, S768I) and *EGFR*-resistant/unknown mutations: from the TCGA cohort, the *EGFR*-sensitive mutant group showed a significantly lower TMB than the resistant/unknown group (median: 60 vs. 283; *p* < 0.001). This was confirmed in a recent study analyzing 153 patients with *EGFR*-mutant lung cancer [96].

Similar results were found in another study in 100 patients from Japan: 10 NSCLC had a high-TMB (>20 mutations/Mb), among whom 2 harbored a driver mutation (1 *ALK* rearrangement and 1 *HER2* mutation), whereas 57 of the 90 specimens with low-TMB harbored an actionable oncogenic driver mutation (*ALK*, *ROS1*, or *RET* rearrangement or *EGFR*, *HER2*, or *MET* mutation) (*p* < 0.05) [97].

**Table 3:** PD-L1 expression in NSCLC with actionable oncogenic driver mutation.

Gene	Study	Population	PD-L1 Status			Ref.
			<1%	≥1%	≥50%	
EGFR	Liu, 2018	EGFR+, all, *n* = 341	78%	22%		[73]
		T790M+, *n* = 32	86%	14%		
		T790M-, *n* = 309	74%	26%		
	Hata, 2017	EGFR+, all, *n* = 67	51%	49%	<1%	[76]
		T790M+, *n* = 26	69%	31%	0%	
		T790M-, *n* = 41	39%	61%	2%	
	Cho, 2018	EGFR+, all, *n* = 319	48%	52%	8%	[74]
		Del19, *n* = 145	48%	52%	6%	
		L858R, *n* = 121	62%	38%	7%	
	Yoneshima, 2018	EGFR+, all, *n* = 70	57%	43%	10%	[89]
		Del19, *n* = 40	50%	50%	13%	
		L858R, *n* = 30	67%	33%	7%	
	Lau, 2020	EGFR+, all, *n* = 17	29%	71%	41%	[79]
		Del19/L858R, *n* = 13	23%	77%	38%	
		Ex20ins, *n* = 4	50%	50%	50%	
	Mazieres, 2019	EGFR+, all, *n* = 49	37%	63%	29%	[83]
	Gainor, 2016	EGFR+, pre-TKI, *n* = 62	76%	24%	11%	[87]
		EGFR+, post-TKI, *n* = 63	69%	31%	14%	
	Karatrasoglou, 2020	EGFR+, *n* = 18	44%	56%	6%	[88]
	Rangachari, 2017	EGFR+, *n* = 13			0%	[92]
	Chen, 2020	EGFR Ex20ins, *n* = 35	51%	49%		[78]
KRAS G12C	Tao, 2020	KRAS G12C, *n* = 40	60%	40%		[91]
MET exon 14	Sabari, 2018	MET exon 14, *n* = 111	37%	63%	41%	[80]
	Mazieres, 2019	MET exon 14, *n* = 20	25%	75%	46%	[83]
	Guisier, 2020	MET exon 14, *n* = 14	8%	92%	79%	[85]
	Dudnik, 2018	MET exon 14, *n*:9	22%	78%	67%	[82]
BRAF	Dudnik, 2018	BRAF, all, *n* = 29	31%	69%	45%	[86]
		V600E, *n* = 19	36%	74%	42%	
		nonV600E, *n* = 10	40%	60%	50%	
	Guisier, 2020	BRAF+, all, *n* = 21	24%	76%	57%	[85]
		V600E, *n* = 14	21%	79%	71%	
		nonV600E, *n* = 7	39%	71%	29%	
	Dudnik, 2018	BRAF+, all, *n* = 13	31%	69%	38%	[82]
		V600E, *n* = 8	25%	75%	25%	
		nonV600E, *n* = 5	40%	60%	60%	
	Mazieres, 2019	BRAF+, *n* = 10	30%	70%	56%	[83]
HER2	Lai, 2018	HER2+, *n* = 87	77%	23%		[84]
	Chen, 2020	HER2+, *n* = 21	81%	19%		[78]
	Mazieres, 2019	HER2+, *n* = 15	47%	53%	0%	[83]
	Lau, 2020	HER2+, *n* = 13	38%	62%	23%	[79]
	Guisier, 2020	HER2+, *n* = 8	50%	50%	13%	[85]
ALK	Gainor, 2016	ALK+, pre-TKI, *n* = 19	37%	63%	26%	[87]
		ALK+, post-TKI, *n* = 12	58%	42%	17%	
	Mazieres, 2019	ALK+, *n* = 11	36%	64%	40%	[83]
	Karatrasoglou, 2020	ALK+, *n* = 11	55%	45%	0%	[88]
ROS1	Dudnik, 2018	ROS1+, *n* = 5	20%	80%	40%	[82]
	Mazieres, 2019	ROS1+, *n* = 5	0%	100%	60%	[83]
RET	Mazieres, 2019	RET+, *n* = 8	25%	75%	50%	[83]
	Dudnik, 2018	RET+, *n* = 8	50%	50%	13%	[82]
	Guisier, 2020	RET+, *n* = 8	62%	38%	25%	[85]

TKI: tyrosine kinase inhibitor.

## 3. Clinical Data on Anti-PD1/PD-L1 Efficacy in NSCLC with Actionable Oncogenic Driver Alterations

Few NSCLC patients with actionable oncogenic driver mutations were included in the pivotal clinical trials evaluating anti-PD1 therapy and the only available data concern *EGFR* (Table 4). A phase 2 trial was initiated to evaluate Pembrolizumab in the EGFR+ population, specifically. Enrollment was ceased for lack of efficacy after the first 11 patients were treated [98]. Only one patient had an objective response, but repeat analysis of this patient’s tumor definitively showed the original report of an *EGFR* mutation to be erroneous.

In a meta-analysis of three trials that compared an anti-PD1/PD-L1 immunotherapy to a second- or third-line chemotherapy with docetaxel, 185 patients had *EGFR*-mutated NSCLC. In this subgroup, there was no benefit of immunotherapy over chemotherapy: HR for OS 1.05 (0.70–1.55) [99].

Of note, combination of anti-PD-L1 therapy with chemotherapy demonstrated some efficacy [100,101]. The IMPOWER 150 trial compared a four-drug regimen with Atezolizumab, Bevacizumab, Carboplatin, and Paclitaxel (ABCP) with ACP and BCP as first-line treatment in stage IV NSCLC. Among patients with *EGFR*-mutated NSCLC (*n* = 79), overall survival was longer in the ABCP arm (not reached), although the difference was not significant (HR 0.61 (0.29–1.28)). Similar results were found in PFS, with a significant advantage to the ABCP regimen over the BCP regimen in the subgroup of patients that were previously treated with EGFR inhibitors (HR 0.42, IC95 (0.22–0.80)). These results suggest that the combination of immunotherapy plus chemotherapy plus anti-VEGF is a promising regimen for patients failing TKIs [100].

Since 2015 and the advent of anti-PD1 in routine practice, some real-world data have been published (Table 3). The largest study of this kind was the ImmunoTarget multicentric worldwide retrospective study [83], which gathered 125 *EGFR*, 43 *BRAF*, 36 *MET*, 29 *HER2*, 23 *ALK*, 16 *RET*, and 7 *ROS1* NSCLC patients treated with anti-PD1 (92%) or anti-PD-L1, mostly in second- (42%), third- (26%) or later treatment lines (27%).

Overall real-world studies show a lack of efficacy of anti-PD1/PD-L1 monotherapy for *EGFR*, *ALK*, and *HER2* subgroups, and mixed results for *RET* and *ROS1* patients, with a lower number of patients reported so far. On the other hand, *BRAF* and *MET* patients had similar benefits of anti-PD1/PD-L1 therapy as compared to patients with no known driver mutation.

Recently, Yamada et al. reported a series of 27 *EGFR*-mutated NSCLC patients treated with anti-PD1/PD-L1 immunotherapy. They showed that uncommon EGFR mutations were associated with a higher response rate and longer PFS than common activating *EGFR* mutations and/or T790M mutation [102]. Two other retrospective studies also reported ORR in exon 20 *EGFR*-mutated NSCLC patients treated with anti-PD1/PD-L1 immunotherapy. In these studies 3/6 and 2/9 EGFR-Ex20ins patients exhibited a tumor response [78,79].

In line with the above-mentioned results of anti-PD1/PD-L1 immunotherapy in *EGFR*- or *HER2*-mutated stage IV NSCLC, a recent retrospective analysis of patients with unresectable stage III NSCLC treated with consolidation durvalumab after definitive chemoradiation reported a shorter PFS in the *EGFR*- or *HER2*-mutated NSCLC patients subgroup (7.5 mo vs. not reached, *p* = 0.04) [103].

**Table 4:** Clinical data on anti-PD1 efficacy in NSCLC with actionable oncogenic driver alterations.

Study		Main Results	Ref.
**Randomized Clinical Trials**			
CheckMate 057	Nivolumab vs. Docetaxel	EGFR (*n* = 82): HR 1.38 (0.69–2)	[61]
		ALK (*n* = 21): no subgroup analysis	
Keynote 010	Pembrolizumab vs. Docetaxel	EGFR (*n* = 86): HR 0.89 (0.45–1.70)	[62]
		ALK (*n* = 8): no subgroup analysis	
OAK	Atezolizumab vs. Docetaxel	EGFR (*n* = 85): HR 1.24 (0.71–2.18)	[63]
		ALK (*n* = 2): no subgroup analysis	
Atlantic (phase II)	Durvalumab	EGFR/ALK (*n* = 107)	[104]
		ORR: 16%, OS: 12.3, PFS 1.9	
IMPOWER 150	AtezolizumabBCP vs. BCP	EGFR (*n* = 79):	[100,101]
		HR for OS 0.61 (0.36–1.03)	
		Subgroup previously treated by TKI (*n* = 50): HR for OS 0.39 (0.14–1.07); HR for PFS 0.42 (0.22–0.80)	
		ALK (*n* = 31): no subgroup analysis	
**Real-world Studies**			
Gainor, 2016	28 EGFR/ALK+ vs. 30 WT	RR 3.6% vs. 23.3%	[87]
Dudnik, 2018	12 BRAF V600E	RR 25%, PFS 3.7 (1.6–6.6)	[86]
	10 other BRAF	RR 33% PFS 4.1 (0.1–19.6)	
Sabari, 2018	24 METex14	RR 17% (6–36), PFS 1.9 (1.7–2.7)	[80]
Rizvi, 2018	17 EGFR, 7 ROS1, 9 BRAF, 2 ALK, 2 RET	Durable clinical benefit in 2 EGFR, 4 BRAF, 2HER2 and 1 ROS1 patients	[64]
Liu, 2018	6 EGFR1 1 ALK	1 EGFR with partial response	[73]
Garassino, 2018	102 EGFR+ vs. 1293 WT	RR 8.8% vs. 19.6% *	[105]
		OS 8.3 vs. 11.0 *	
Wei-Chu, 2018	26 HER2	RR 12%, PFS 1.9, OS 10.4	[84]
Mazieres, 2019	125 EGFR	RR 12%, PFS 2.1	[83]
	43 BRAF	RR 24%, PFS 3.1	
	36 MET	RR 16%, PFS 3.4	
	29 HER2	RR 7%, PFS 2.5	
	23 ALK	RR 0%, PFS 2.5	
	16 RET	RR 6%, PFS 2.1	
	7 ROS1	RR 17%	
Morita, 2019	116 EGFR	OS 12.1 vs. 14.6 * PFS 1.5 vs. 2.3 * RR 8.6% vs. 22.6 *	[106]
Bylicki, 2020	42 EGFR	OS 13.9 (8.8–20), PFS 2.2 (1.4–3.2)	
	8 ALK	OS 19.2 (13.1-NR), PFS 2.4 (2.1-NR)	
	1 ROS1	OS 2.8, PFS 1.4	
Barlesi, 2020	44 EGFR	OS 8.1 vs. 12.2	[107]
Guisier, 2020	26 BRAF V600	RR 26%, PFS 5.3, OS 22.5	[85]
	18 BRAF NV600	RR 35%, PFS 5.3, OS 12	
	30 MET	RR 36%, PFS 4.9, OS 13.4	
	23 HER 2	RR 27%, PFS 2.2, OS 20.4	
	9 RET	RR 37%, PFS 7.6, OS NR	
Lau, 2021	28 EGFR SM	RR 11%, PFS 1.7,	[79]
	6 EGFR-Ex20ins	RR 50%, PFS 4.8,	
	14 HER 2	RR 29%, PFS 3.6	
Chen, 2021	9 EGFR-Ex20ins	RR 22%	[78]
	6 HER2-Ex20ins	RR 0%	
Yamada, 2021	20 common EGFR	RR 10%, PFS 1.6	[102]
	7 uncommon EGFR	RR 57%, PFS 8.5	

BCP: Bevacizumab + carboplatin + paclitaxel, SM: sensitizing mutations, WT: wild-type, RR: response rate, PFS: progression-free survival, OS: overall survival. PFS and OS are given in months. * comparisons are shown between *EGFR*-mutated and *EGFR* wild type NSCLC patients.

## 4. Future Directions

Use of anti-PD1/PD-L1 monotherapy in NSCLC harboring common *EGFR* mutation or *ALK* rearrangement can be ruled out as a standard strategy given the bad outcomes of patients treated in this setting. After EGFR/ALK TKI failure, the combination of chemo-immunotherapy with an antiangiogenic agent is under investigation (NCT04042558) and may improve outcomes over chemotherapy alone or combined with an antiangiogenic agent.

In *KRAS* or *BRAF* mutated NSCLC, anti-PD1/PD-L1 immunotherapy exhibits high efficacy. As more targeted therapies are developed in this setting, the question is now to evaluate the best sequence and/or combination of treatments. *KRAS* G12C inhibitors sotorasib and adagrasib have a favorable safety profile that may allow combination with anti-PD1/PD-L1 treatment, a strategy that is under investigation for first-line treatment (NCTXXX). *BRAF* V600E inhibition with anti-BRAF and anti-MEK inhibitors is associated with more toxicities, which may preclude their use in combination with anti-PD1/PD-L1 agents. Comparison of first-line treatment with TKIs or chemo-immunotherapy is needed in this setting. The same question is arising for *MET* and *HER2* mutated as new targeted therapies are being developed and reach first- or second-line treatment.

For other rare targetable drivers, data is too scarce to draw definitive conclusions about the place of anti-PD1/PD-L1. Gathering large cohorts of patients in this setting is challenging but collaborative efforts are ongoing such as the RET-MAP study. 

## 5. Conclusions

NSCLC with driver mutations represent a challenging population for the clinician as large clinical trials often do not take into account the particular biology of these subgroups. Preclinical data are useful for evidence-based decisions, but real-world studies are particularly important to assess their relevance. Network efforts to gather large cohort should be encouraged in this perspective.

Anti-PD1/PD-L1 therapy has been a revolution in the field of advanced NSCLC, notably by improving the prognosis of stage IV disease. It gave rise to a whole new population of patients, the long-term survivors, who did not exist in that setting before the immunotherapy era. Nevertheless, here we showed that some subgroups of patients do not derive a benefit from these drugs, particularly patients with *EGFR*- or *HER2*-mutated or *ALK*-rearranged NSCLC. On the other hand, *BRAF*- and *MET*-mutated NSCLC seem to be as sensitive to anti-PD1/PD-L1 immunotherapy as unselected NSCLC. Patient selection using validated biomarkers and inclusion in clinical trials are key to improve their outcome. Biomarker studies beyond PDL-1 expression are needed and achievable in *EGFR*, *ALK*, *BRAF*, *HER2*, *RET*, *NTRK*, *KRAS G12C*, and *MET*-mutated NSCLC patients.

## Figures and Tables

**Table 1:** Actionable oncogene alterations in NSCLC and corresponding targeted therapies.

Gene Alteration	Freq.	Targeted Therapy	Ref.
*EGFR* activating mutations	15–50%	Erlotinib	[10]
		Gefitinib	[11]
		Afatinib	[12]
		Dacominib	[13]
		Icotinib	[14]
		Osimertinib	[15]
		Mobocertinib	[16]
		Poziotinib	[17]
*ALK* rearrangement	4%	Crizotinib	[18]
		Ceritinib	[19]
		Alectinib	[20]
		Brigatinib	[21]
		Lorlatinib	[22]
*MET* exon 14 skipping mutations	4%	Crizotinib	[23,24]
		Cabozantinib	[25]
		Capmatinib	[26]
		Tepotinib	[27]
		Savolitinib	[28]
*BRAF* mutations	3%	Vemurafenib	[29]
		Dabrafenib	[30]
		Dabrafenib + Trametinib	[31]
*HER2* mutations	3%	Trastuzumab	[32]
		Neratinib	[33,34]
		Afatinib	[35]
		Lapatinib	[36]
*ROS1* rearrangement	1–2%	Crizotinib	[37]
		Ceritinib	[38]
		Lorlatinib	[39,40]
		Entrectinib	[41]
*RET* rearrangement	1–2%	Vandetanib	[42]
		Cabozantinib	[43,44]
		Pralsetinib	[45]
		Selpercatinib	[46]
*NTRK* fusion	<1%	Entrectinib	[47,48]
		Larotrectinib	[48]
		Selitrectinib	[49]
*Kras G12C mutation*	13%	Sotorasib	[50]
		Adagrasib	[51]
		Adagrasib	

Freq.: percentage among non-squamous NSCLC [8,9,52].
